# How can data visualization support interdisciplinary research? LuxTIME: studying historical exposomics in Belval

**DOI:** 10.3389/fdata.2023.1164885

**Published:** 2023-09-29

**Authors:** Dagny Aurich, Aida Horaniet Ibañez

**Affiliations:** ^1^Luxembourg Centre for Systems Biomedicine, University of Luxembourg, Esch-sur-Alzette, Luxembourg; ^2^Luxembourg Centre for Contemporary and Digital History, University of Luxembourg, Esch-sur-Alzette, Luxembourg

**Keywords:** data visualization, historical data, interdisciplinary research, exposome, digital history

## Abstract

The Luxembourg Time Machine (LuxTIME) is an interdisciplinary project that studies the historical exposome during the industrialization of the Minett region, located in the south of Luxembourg. Exposome research encompasses all external and internal non-genetic factors influencing the health of the population, such as air pollution, green spaces, noise, work conditions, physical activity, and diet. Due to the wide scope of the interdisciplinary project, the historical study of the exposome in Belval involved the collection of quantitative and qualitative data from the National Archive of Luxembourg, various local archives (e.g., the communes of Esch-sur-Alzette and Sanem), the National Library, the Library of National Statistics STATEC, the National Geoportal of Luxembourg, scientific data from other research centers, and information from newspapers and journals digitized in eluxemburgensia.^1^ The data collection and the resulting inventory were performed to create a proof of concept to critically test the potential of a multi-layered research design for the study of the historical exposome in Belval. The guiding navigation tool throughout the project was data visualization. It has facilitated the exploration of the data collected (or just the data) and the metadata. It has also been a valuable tool for mapping knowledge and defining the scope of the project. Furthermore, different data visualization techniques have helped us to reflect on the process of knowledge sharing, to understand how the relevance of certain topics changed throughout the project and why, and to learn about the publication process in different journals and the experience of the participants. Data visualization is used not only as a means to an end but also to embrace the idea of *sandcastles* using a speculative and process-oriented approach to advance knowledge within all research fields involved. LuxTIME has proven to be an ideal case study to explore the possibilities offered by different data visualization concepts and techniques resulting in a *data visualization toolbox* that could be evaluated and extended in other interdisciplinary projects.

## 1. Introduction

Interdisciplinary collaboration, especially in projects involving researchers across the natural and applied sciences and the humanities, presents many challenges, including the multiplicity of research questions, the use of different methodologies, and the varying underlying assumptions based on different epistemic cultures. Bridging these epistemological and methodological differences to establish a shared understanding requires a substantial intellectual commitment from all participants. In an ideal scenario, such collaboration has the potential to produce new research questions, foster interdisciplinary approaches for the analysis of complex issues, and generate new knowledge based on interactional expertise (Fickers et al., 2022). Data visualization can play a significant role in facilitating such interdisciplinary collaboration.

Data visualization is widely used in research for exploring quantitative data, validating hypotheses, and communicating results, primarily using statistical charts such as bar charts, line charts, or scatter plots (Glivinska, 2021). It is less frequently used to study the research process itself. It is a process through which ideas are explored and a collective discourse is constructed using data visualization to critically observe different mediations (Hinrichs et al., 2019). In this article, we reflect on some of the data visualizations designed during the Luxembourg Time Machine (LuxTIME) project discussed in section 2. Based on the experience obtained during 2.5 years of interdisciplinary collaboration, working with a team of historians, hydrologists, chemists, and data visualization experts, we want to demonstrate that interdisciplinary research can benefit from an “extended” *data visualization toolbox*. We refer to a *toolbox* as a set of data visualization concepts and techniques, and we want to “extend” it, as opposed to only applying techniques frequently used within each specific field.

Specific disciplines can develop certain data visualization conventions. Considering visualization in interdisciplinary projects can help break out of these conventions and create new insights. Statistical graphs are used mostly in the natural, social, and applied sciences with the objective of exploring data to discover trends, patterns, and outliers, and to validate hypotheses or communicate results quickly for decision-making. Visualizations that emphasize esthetics and use metaphors and non-standard visual vocabularies are often found in journalistic, educational, and artistic contexts, while the study of interpretive practices and non-representative approaches predominate in the humanities. Each discipline can benefit from different practices. This becomes even more evident in the case of interdisciplinary research.

Combining these concepts and techniques predominant in different disciplines and applying them in the same context, we developed the *LuxTIME data visualization toolbox* exploring standard statistical graphs and variations thereof, concept maps, visual rhetoric, data humanism, multivariate data glyphs, non-representational approaches and visual elements of interpretation, and data storytelling. The use of different types of visualizations helped to map and exchange knowledge, thereby defining the project scope and the contributions of different disciplines, track timelines, and project deliverables. It also framed the participants' experiences along the way, inviting them to self-reflect on changes throughout the project and to explore the iterative research process. The *toolbox* aims to inspire a wide range of projects, especially those involving different disciplines. It describes and discusses the application of a set of epistemologically distant techniques and concepts from which to begin the exploration, and then add or remove tools, adapting the *toolbox* to each project. Foremost, it extends an invitation to explore and integrate other ways to visualize data, diverging from the traditional techniques commonly employed in each respective discipline.

In the current article, we start with a description of the LuxTIME project. Next, we discuss the methods, including the data collection process, the participants and their roles, the research questions addressed in the interdisciplinary context, the visualization concepts and techniques included in our project, and their application in the results section. We finish with a discussion of the frictions experienced as part of working on an interdisciplinary project with data visualization, how the idea of data visualization as a *sandcastle* (Hinrichs et al., 2019) has been present throughout the project, and finally, how other projects can benefit from our experience.

## 2. Case study

The Luxembourg Time Machine Project (LuxTIME) is an interdisciplinary research project funded by the Institute for Advanced Studies (IAS) of the University of Luxembourg.^2^ Three research institutes engage in this so-called “Audacity Project”, namely, the Center for Contemporary and Digital History (C^2^DH), the Luxembourg Center for Systems Biomedicine (LCSB), and the Luxembourg Institute of Science and Technology (LIST). The main objective is to explore the potential implementation of a national platform (“Luxembourg Time Machine”) that would allow scientists and stakeholders to “dive” into the complex past of this country using digital tools and data from different disciplines and fields. It is a subsidiary project of the European Time Machine project^3^ adding a new dimension to the past.

By building a digital dataset including information from three very different fields and scientific perspectives, namely, eco-hydrology, environmental cheminformatics, and history, LuxTIME is using a local case (the industrialization of Belval and the Minett region) as a testbed for methodological and epistemological reflections on how to study the impact of environmental changes on the health of the local population, with a regional and long-term perspective. “Contextual information” based on archival evidence is mixed with “scientific evidence” derived from chemical, biological, or medical investigations as the project explores new grounds in interpreting “big data of the past” in a truly interdisciplinary setting. The Belval case study is a pilot project, preceding a project at the national level, the LuxTIME INITIATE.^4^

The above-mentioned impact of environmental changes on human health is covered by the exposome concept (Miller, 2020). Global and local changes have severe impacts on environmental systems and their inhabitants (Karlsson et al., 2021). The analysis of those changes and their impacts is a challenging task that can, however, be of help to understand and therefore prevent potential future outcomes. This could be, for example, any case of environmental pollution happening in an area resulting in an increase in disease cases as a phenotypic response. Human phenotypes, being a set of observable characteristics or traits, are mainly influenced by two factors and their interactions: the genetic factors described by the *genome* and all non-genetic factors covered by the *exposome* concept. Measuring the genome is difficult, but it is limited to the combination of four nucleotides that are stable over time. The *exposome* of an individual, however, is the measure of environmental influences (e.g., lifestyle, diet, and behavior) and the associated biological responses (Miller and Jones, 2014), changes within the course of a lifetime, and historical developments. Consequently, the *exposome* is influenced by many factors that vary over time and influence each other. Interdisciplinary efforts and data sources are required to come as close as possible to covering the whole picture.

The focus of this article was on how the use of a variety of data visualization concepts and techniques has supported our interdisciplinary (exposome) research.

## 3. Methods

### 3.1. Data collection

In this article, we refer to *data* as any collection of values conveying information, whether to represent abstract ideas (e.g., knowledge exchange and relevance of a topic), specific measurements (e.g., concentration of chemicals and number of articles published), or statistics (e.g., census data and steel production). We collected quantitative and qualitative data, mostly from secondary data sources (e.g., statistical archives, existing research), but also included some primary sources (e.g., observations from historical sources and reflections on the process itself). The collection for the LuxTIME project was done through observation, measurement, simulation, and analysis.

The Minett region is known for its past in the iron and steel industry, which was accompanied by many regional changes in terms of environment, socioeconomy, and health (Knebeler and Scuto, 2010). Some of the initial questions of the LuxTIME members included the following: where to look for data (*e.g.*, local and national archives); where to set the geographical and time limits; which topics to focus on (*e.g.*, environmental pollution); and how to obtain scientific data. For example, in the case of performing new chemical analyses, which sample types could be used? The initial objective was to find links between environmental pollution and other influencing factors of the past and disease patterns in the population by looking at archival sources combined with information received by scientific or governmental institutes and current chemical analyses, revealing facts about past exposures. This includes information related to the *historical exposome* in Belval such as datasets, images, text, events, and maps. For each dataset, relevant metadata was also collected, including title, source, reference from the source if available, author, publication date, the team member who collected the information, language(s), description, number of files, digitization status, class, format, type, period covered, geographical area covered, access rights, and the categories and subcategories of the *exposome* covered. Furthermore, we collected information about the process, such as the relevance of the different topics, disciplines involved, period of validity and reasons for change, relation to other disciplines, the amount of information found, and its potential for further analysis. We also collected data about the project deliverables such as the number of publications, the type, the disciplines involved, the different steps in the process (e.g., work starts, submission, and publication), the time, and the experience.

All these quantitative and qualitative data allowed us to study the research questions stated in section 3.3, using two data collection methods. First, a structured and normalized *data inventory* was created, where we included all the pieces of information found through the sources. To date, this table contains 121 records from 17 different information sources, each of them registered with the metadata described above. Second, for the evolution, reflection on the process, and experience, we used the visualization directly to generate the data (that could be extracted later if necessary).

The first steps included contacting governmental and scientific institutes to access past data already collected in the area (*e.g.*, scientific measurements of parameters such as soil or air pollution resulting from industry or other anthropogenic sources). Moreover, measures taken by the industry or the government to enhance life quality and health were investigated. Historical data—not only about environmental pollution but also social and economic data—were retrieved from archives. Newspaper articles, scientific reports and books, pictures, and contemporary witness reports were included. For chemical measurements generating scientific data, sampling campaigns of surface and groundwater were discussed as well as looking at dust samples and soil or biological samples such as mussels, trees, or even teeth. Based on the research on this topic from a chemistry point of view, a review article was published in 2021 (Aurich et al., 2021), helping to plan further project work packages in terms of, e.g., sampling campaigns. One example is the discussion of analyzing human samples to get to know more about past steel-pollution exposure and its health effects. However, the sample bank located in Luxembourg does not provide samples dating back to the steel industry times as it is a fairly new facility. The outcomes of the review showed many possibilities for how to access the historical *exposome* in terms of data and chemical analyses; however, none of them was available or feasible within the project scope and timeline.

### 3.2. Participants and roles

The core of the research described in this article was conducted during the collaboration between a researcher in data visualization and a researcher in environmental cheminformatics, with occasional feedback and participation from the overall project team that included other researchers in environmental cheminformatics, eco-hydrology, and history. The researcher in environmental cheminformatics already had disciplinary knowledge of data visualization and functioned as a domain expert in the field of the *exposome*, together with the rest of the environmental cheminformatics team. The data visualization researcher collected and studied many techniques and concepts used across different disciplines and then proposed an initial *toolbox* to discuss and experiment with during the visualization sessions are described in section 3.5. The researcher in data visualization also participated in the work of the target domain, *historical exposomics*, and the environmental cheminformatics researcher participated in visualization research. The collaboration was based on a *design-by-immersion* approach with reciprocal immersion, where both the visualization researcher and domain expert engaged with and participated in the work of the other domain, and knowledge emerged from the experiences and interactions (Hall et al., 2020). They not only participated in each other's research approaches and practices but also did archival work, collecting and analyzing historical sources. As discussed in section 5, this approach shapes and enriches the research team and also changes both participants' perspectives on their own fields.

### 3.3. Research questions

With data visualization as a navigation tool, our goal is to analyze the research questions outlined below.

*Question #1: How could we map knowledge and knowledge sharing to define the project scope?* The first question aims at exploring the *knowledge gap*, resulting from the different backgrounds and expertise in the project, e.g., history, design, and chemistry (Van Wijk, 2006). To create a “trading zone”, i.e., “a space for interactions and negotiations between different knowledge domains” (Fickers and van der Heijden, 2020), we first needed to understand the knowledge within the group and what needed to be shared to define the project scope. In this research question, we explored how to arrive at an initial space, which exists at the boundary of four disciplines in which research can begin; a space in which diverse voices can speak and be heard and differences can be examined to mutually validate diverse perspectives, creating opportunities for mutual learning (Mao et al., 2019).

*Question #2: How did the project evolve in terms of scope, relevance of the topics, the information available, and blending of the disciplines in each of these topics?* The scope of the project was not permanent, nor was the knowledge or interest of the different disciplines in the different topics, which blended with different intensities throughout the process; as the project progressed, new sources of information appeared, and others were discarded. In this research question, we were interested in the evolution of how, from a series of central themes defined in the previous question, the scope changed throughout the project: which topics gained importance, which ones appeared or disappeared, and why.

*Question #3: How could we explore the data and metadata respecting the priorities of the different disciplines?* To synthesize disparate datasets in a visualization, especially in a project involving epistemologically distant disciplines, *data frictions* emerge regarding discipline-specific interpretation of the data, methodological approaches, ways of handling uncertainty, and scale and granularity in the datasets (Panagiotidou et al., 2022). Without analyzing in detail the different causes of such frictions, in this research question, we explored how the use of visualization techniques from other disciplines reveals the different priorities, and how through co-construction and exchange throughout the process, a common space of data and metadata visualization can be reached.

*Question #4: How could we monitor the project deliverables, including the different steps of the process, the contribution of the different disciplines, and the experience of the participants?* In addition to the *knowledge gap* discussed in the first research question, there is often an *interest gap*, caused by the different aims of the researchers, e.g., participation in different types of conferences and publication requirements (Van Wijk, 2006). These differences not only have an impact on the deliverables and, therefore, on the project timeline but also on the experience of the participants. In this research question, we explored the representation of the different steps in the process in time vs. in the experienced temporality.

We have explored each of these questions through visual means. In section 3.4, we introduce the concepts and techniques that we have included in our *toolbox* throughout the project followed by a discussion of the visualizations in which they are applied, in section 4.

### 3.4. The LuxTIME data visualization toolbox

We refer to *data visualization* as the graphical representation of data, using a variety of visual encoding methods (a representational approach), as well as the use of a graphical representation, to model interpretation and generate or augment data (a non-representational approach). The applications vary (e.g., exploratory analysis, data validation, hypothesis validation, and communication), and the techniques used depend on the intended purpose (e.g., quick decision-making vs. in-depth exploration of multiple narratives). Data visualization (or Dataviz) encompasses other terms including information visualization (InfoVis), information design, scientific visualization (SciVis), information graphics (Infographics), statistical graphics, or exploratory data analysis. The difference between these terms has been largely discussed in previous research (Rhyne et al., 2003; Manovich, 2011; Lankow et al., 2012; Kim et al., 2016).

In this section, we discuss several data visualization concepts and techniques that have been fundamental to our project and, therefore, essential elements of our *data visualization toolbox*.^5^ The selection of “tools” for our *toolbox* is based on an extensive literature review and the study of numerous data visualization examples from different disciplines. It aims at integrating the perspectives on the field of the different disciplines (discussed below in relation to each concept) and above all to experiment with concepts and techniques originating from epistemologically distant disciplines, which are rarely applied in the same context. The *toolbox* is built with an inclusive approach, integrating visualizations frequently used across disciplines (e.g., statistical charts) and potential variations, which also led to rich conversations and outcomes.

Some of these elements may be useful for other interdisciplinary projects; therefore, we present below a brief description and discussion of each of them, including examples from other projects. These methods and techniques are then applied in section 4 to answer our research questions.

#### 3.4.1. Statistical graphs

The most frequently used data visualization techniques across disciplines are all types of statistical charts, such as bar charts to compare magnitudes, line charts to show evolution in time, or scatter plots to analyze relationships (Glivinska, 2021). The main purpose of these graphs is to summarize, validate, and communicate a message effectively. The use of statistical graphs assumes that the audience is data literate, i.e., has some basic knowledge of descriptive statistics; and that the designer^6^ is aware of the extensive existing research on the subject.

Statistical graphs have a long history, and there is extensive literature about how to use them correctly to explore and summarize data effectively. However, academic and non-academic data visualization practitioners from different disciplines often fail to apply these theoretical principles. One of the frequent pitfalls is the wrong selection of charts despite the literature about graphical perception and suitability for analytical purposes (Lockwood, 1969; Cleveland and McGill, 1984, 1986; Evergreen, 2017). Other errors include the inappropriate use of colors based on color perception or cultural differences (Rogowitz and Treinish, 1998; Ware, 2004; Silva et al., 2007, 2011), the use of misleading graphs (Cairo, 2019), or poor storytelling (Nussbaumer Knaflic, 2015; Dykes, 2020). Moreover, the frequent lack of polish on elements such as axes, labels, gridlines, annotations, legends, descriptions, and titles (Schwabish, 2021) can create distractions from the core message (Tufte, 1999). These are just a few examples among many other considerations to be examined when creating statistical graphs (e.g., layout, context, transparency, accessibility, and interactivity).

Applied cases of statistical graphics to the LuxTIME are demonstrated in sections 4.3.1 and 4.3.2.

#### 3.4.2. Variations of statistical graphs

We refer to the variation of a statistical graph when, knowing the theory mentioned in the preceding section, the designer decides not to apply one or more of these rules deliberately, e.g., to meet a specific use case requirement. Examples of such variations include duplicating the encoding (i.e., overencoding) to highlight a particular aspect of the graph (e.g., position and color encode the same information), changing the orientation, or overlapping graphs to favor a particular visual effect (e.g., crowdedness). Other options include removing axes, measures, gridlines, or titles to focus the audience's attention on the visualization; replacing predefined geometric shapes with other elements with a rhetorical value (e.g., using the representation of an object instead of rectangles in a bar chart); or any other type of visualization that starts from a statistical graph and modifies it with a purpose, beyond the one initially established for such a graph. It is often a combination of several modifications. Such variations of the conventional statistical graphs open numerous possibilities for the designer, especially in terms of communication.

The *climate stripes* by Ed Hawkins are a variation of a statistical graph, showing a simplified heatmap that has a strong influence on the climate change debate around the world. ShowYourStripes.info^7^ registered 89,000 unique visitors to the site worldwide for 30 days during the summer of 2022 (Santoro and Kirkland, 2022). Hawkins emphasized the need for a range of ways of communicating the “climate crisis” because different people learn and experience in different ways and, therefore, justified the need for a range of climate visualizations to talk to different audiences. Another example is the visualization of China's overseas investments by Alberto Lucas López and Cédric Sam^8^ where the bars of a bar chart are replaced by semicircles whose area represents the value of the deals and is displayed for many countries, overlapping in the layout. Laura Bronner, Anna Wiederkehr, and Nathaniel Rakich visualized the election night in 2020 *What Blue And Red “Shifts” Looked Like In Every State*^9^ using simplified area charts in a tile map of the United States for FiveThirtyEight.^10^ Kim Albrecht explores the randomness of success in scientific publications^11^ using overlapping timelines in which only the maximums are highlighted. Another visualization example by Alberto Lucas López in collaboration with Ryan Williams and Kaya Berne is *Migration waves*,^12^ where variations of area charts are used, through minimalism (no grids, no axes, and no measures), color (to emphasize positive and negative currents), and the superimposition of the graphs for the different countries.

#### 3.4.3. Concept maps

Concept mapping is a visualization technique that uses hierarchical networks of nodes (concepts) and links (relationships) to represent visual knowledge (Romance and Vitale, 1999). The use of a concept map allows the inclusion of cross-links to map the links between concepts in different domains and represent creative leaps in knowledge production (Novak and Cañas, 2008). Joseph D. Novak and Alberto J. Cañas identified two features of concept maps that are important in the facilitation of creative thinking: the hierarchical structure and the ability to search for and characterize new cross-links. In addition to the purpose of enhancing creativity, concept maps are used as a design tool to generate structural organization, for communication purposes to overcome the limitations of the linear nature of the text, to stimulate the learning process by making the interrelationships between concepts explicit, and as an assessment tool to identify misconceptions (Lanzing, 1998). Johannes Wheeldon and Jacqueline Faubert argued that traditional definitions of concept mapping should be expanded to include more flexible approaches to the collection of graphic representations of experience, using concept maps to gather qualitative data from research participants (Wheeldon and Faubert, 2009).

In an interdisciplinary context where mapping knowledge of the different stakeholders is key to understanding the possibilities and developing the project, concept maps are a data visualization technique that helps to navigate the complexity, in terms of the variety of topics and subtopics and how they interrelate (see applied for LuxTIME in section 4.1).

#### 3.4.4. Visual rhetoric

Rhetoric is the study of the communication techniques used to inform, persuade, or motivate a given audience in a particular situation, modifying their conceptions and attitudes toward the object of communication. Visual rhetoric (as an artifact) is the purposeful production or arrangement of colors, forms, and other elements to communicate with an audience (Hill and Helmers, 2009). One case of visual rhetoric in the context of data visualization is mapping rhetoric, which refers to “manipulating the information presentation via the data-to-visual transfer function, the constraints that determine how a piece of information will be translated to a visual feature” (Hullman and Diakopoulos, 2011).

A metaphor is a rhetorical figure, which refers to the cognitive process humans engage in when they reconceptualize a concept from a target domain in terms of another; and, therefore, when visual language is used to perform these functions, it is a visual metaphor (Steen, 2018). Despite the predominance of a minimalist data visualization approach that favors high data-ink ratios,^13^ the data visualizations that use visual rhetoric—to engage, communicate, and be memorable using visual metaphors, and elements of embellishment—are still very present, especially in the fields of journalism, information design, or data art. Often these types of visualizations use organic forms, associated with nature, such as plants or rocks. Lima (2014) devoted an entire book to the study of trees in visualization, where he analyzes how throughout history, their trunks, branches, leaves, and fruits have served to represent connections between entities, through different domains of knowledge (e.g., family trees, systems of law, and biological species like trees, see application to LuxTIME in section 4.2).

An example of mapping rhetoric is *What's Cookin?*^14^ by Sarah Emery Clark, where she used the metaphor of preparing a meal to explore and analyze the state of the data visualization industry. Other examples of visual rhetoric include the visualization *Apparel Exports to the US* by Liz Bravo, where she visualized trends in the clothing industry using area charts shaped as sewing patterns; One *Angry Bird* by Periscopic,^15^ displaying emotional arcs of the past 10 U.S. presidential inaugural addresses; or *The Great War* by Valentina D'Efilippo,^16^ visualizing the fatalities during World War I as a poppy field. In *A View on Despair*,^17^ Sonja Kuijpers visualized suicide in the Netherlands in 2017, representing the different categories in a landscape.

#### 3.4.5. Data humanism

In *Data Humanism: The Revolutionary Future of Data Visualization*, Giorgia Lupi advocated for the connection of numbers to knowledge, behaviors, and people as data represent real life; making data unique, contextual, and intimate (Lupi, 2017). To do so, she promotes embracing a certain level of visual complexity, i.e., high-density data visualizations containing multiple attributes; and moving beyond standards, away from conventional graphics, and out-of-the-box solutions, to expand the “data-drawing vocabulary”. She stated that “data is a tool that filters reality in a highly subjective way” and, therefore, it is important to reclaim a personal approach to how data are captured, analyzed, and displayed, as data are imperfect. She urged a paradigm shift to “always sneak context in”, in which data visualization embraces imperfection and approximation, “allowing ways to use data to feel more empathetic, to connect with ourselves and others at a deeper level”.

In their book *Data Feminism*, Catherine D'Ignazio and Lauren F. Klein stated that “refusing to acknowledge context is a way to assert authoritativeness and mastery without being required to address the complexity of what the data actually represent” (D'Ignazio and Klein, 2020). Based on the concept of “situated knowledge”, initially raised by Donna Haraway in the 1980s, they stated that the responsibility of ensuring that the situatedness of data is considered is with the person evaluating the knowledge or building upon it. Yanni A. Loukissas referred to errors in data collection as “signifiers taken out of their original interpretative texts” (Loukissas, 2019). Hannah Schwan, Jonas Arndt, and Marian Dörk (Schwan et al., 2022) identified key aspects of disclosure, i.e., the aspiration to be conscious of the potential effects of the designer's assumptions. They invited “the viewer into exchanges with the designer, reflections about the visualization, and engagement with an issue” (Dörk et al., 2013) and proposed several representation forms to integrate the disclosure information into the visualizations.

Some examples of visualizations that use information-rich designs and custom visual vocabularies, and highlight the relevance of details and the imperfection of the data include *Data Items: A Fashion Landscape*,^18^ visualizing the role of fashion connecting people and cultures, and *Bruises—The Data We Don't See*,^19^ depicting a sensorial picture of a personal journey with a disease, both by Giorgia Lupi. In *Trending seeds*,^20^ Valentina d'Efilippo and Lucia Kocincova analyzed and visualized the Twitter social movement #MeToo to understand if social media could become a vehicle to foster social change and reshape traditional views. In *Data Selfi*,^21^ Kadambari Komandur explored intersectional feminism. These are just a few examples among many others. Moreover, the concept of data humanism has been discussed in research articles over the last few years. Kim et al. (2018) presented an interface that enables designing and personalizing visual vocabulary to represent data, and they explored how to enable people to determine the representation of their data based on the *Dear Data* project (Kim et al., 2019).^22^ Cordell advocated for exploratory, iterative, and dialogic data humanism to foster humanistic engagement with data in an academic context (Cordell, 2019). This concept is applied in several visualizations in section 4.

#### 3.4.6. Multivariate data glyphs

A data glyph is a visual representation of data where the attributes of the graphical entity are defined by the attributes of the data record. It is a visualization technique often used for multivariate data because patterns involving more than two dimensions can often be perceived more easily in this manner (Ward, 2008). Multivariate or multidimensional data consist of a list of records, with multiple columns (variables), which may be either numerical or categorical values. The encoding can map one data attribute to one single graphical attribute; or use redundant mappings (e.g., using tone and shape to display the same variable) to facilitate the interpretation or reinforce a message. Small multiple data visualizations contain several small graphs arranged on a grid, where every representation follows the same structure; and leverages visual constancy, economy of perception, and uninterrupted visual reasoning (Chuah and Eick, 1998). Small multiples can be based on conventional graphs, but also on information-rich glyphs that encode data attributes using customized visual vocabularies. Glyphs can be displayed in a layout based on data variables, data structure (e.g., time and hierarchies), or any other layout (e.g., predefined shape and screen size). The use of glyphs allows the designer to define the level of aggregation, where each glyph is the level of detail selected. They are often used to explore details (e.g., people, objects, and cases) as they allow us to visualize multiple characteristics about each subject (see applied in section 4.3.1).

*Take a walk down Fifth Avenue*^23^ by Molly Morgan uses data glyphs to represent the physical characteristics and the ecological benefits of every tree along Fifth Avenue in New York. Alberto Lucas López in *Our daily faces*^24^ depicts every page of the South China Morning Post newspaper over a year, encoding the subjects covered or the length in small glyphs. *Representation of women in politics*^25^ by Frederica Fragapane visualizes the top 40 countries in the world by political parity score, using a combination of graphical attributes to represent multiple variables for each country, such as the percentage of seats held by women in local government bodies, in lower and upper houses of national legislatures; the number of female candidates in the most recent elections; the number of elected or appointed heads of state or the geographical area.

#### 3.4.7. Non-representational approaches and interpretation

Inspired by the work of Thrift (2008), Johanna Drucker defined non-representational approaches to modeling interpretation in a graphical environment “as the use of graphical means as a primary method of modeling human-authored interpretation rather than to display preexisting data sets” (Drucker, 2018). In contrast to representational approaches, the existence of data or other representations is not assumed before the interpretative work; the relationship between data and visualization is not unidirectional. Visualization can be the starting point, where we add, for example, connections or annotate reflections and use any visual vocabulary to encode high-level concepts (e.g., contradiction and comparison). This could be later captured as data. As already discussed in previous sections, situated, experiential, and embodied forms of knowledge have been largely researched (in contrast to observer-independent empirical approaches). “A humanistic approach is centered in the experiential, subjective conditions of interpretation” and, therefore, it requires a shift toward non-standard metrics, where the challenge is to design graphical expressions that display interpreted phenomena (Drucker, 2011). Time, for example, is modeled not to imitate its physical dimension but to provide a model that reflects the phenomena under consideration to support a given set of analyses (Aigner et al., 2011). Conventional approaches to timelines are linear, unidirectional, continuous, and structured with a single standard metric unit because they take their structure from the temporal models used in the natural sciences. In humanities, temporality is experienced as asynchronous, variable, broken, and heterogeneous (Drucker, 2021).

There are several examples of data visualizations being used as the starting point to collect and visualize data, for example, in participatory projects using street data walls such as the *Mood Test*^26^ by Domestic Data Streamers to analyze people's attitudes toward life; or in data physicalization projects such as the *Data Badges* (Panagiotidou et al., 2020) that invited participants of a conference to make their own customized expressions of their academic profiles. However, examples of interpretive exercises through visualization where graphical attributes are designed to show subjective conditions of interpretation are practically non-existent, beyond the theoretical study presented above and some tool prototypes such as the 3DH project.^27^

When we want to approach analysis from a more humanistic perspective, for example, to analyze an experience, the use of a non-representational approach to data visualization using standard and non-standard metrics, as required, can facilitate the interpretative exercise. In the case of the LuxTIME project, we use this technique to reflect on the evolution of the project's topics in section 4.2 and to understand the participants' experience in section 4.4.

#### 3.4.8. Data storytelling

Data storytelling is a powerful mechanism for sharing insights that involve data, narrative, and visuals to explain, i.e., narrative couples with data, explain; visual couples with data, enlighten; and narrative coupled with visuals, engage (Dykes, 2020). Storytelling makes data interesting, facilitates the understanding of complex subjects, encourages action, and is memorable (Vora, 2019). Brent Dykes identifies nine main tasks as part of the storytelling process: identifying key insights, being aware of biases, having extensive background knowledge, understanding the audience, curating the information, assembling the story, providing narration, choosing the visuals, and adding credibility (Dykes, 2020). Edward Segel and Jeffrey Heer developed a framework of design strategies for narrative visualization in the context of journalistic storytelling, where they placed the visualizations along a spectrum of *author-driven*, i.e., linear structure, heavy messaging, and no interactivity, and *reader-driven* approaches, i.e., highly interactive with no clear path to the story (Segel and Heer, 2010). “Narrative information visualizations rely on rhetorical techniques, to convey a story to users as well as exploratory, dialectic strategies aimed at providing the user with control over the insights gained from interaction.” (Hullman and Diakopoulos, 2011).

*Framing Luxembourg*,^28^ a timeline tracing the history of public statistics in Luxembourg, is an example of data storytelling, where the narrative is divided into different chapters (e.g., migration, family, and employment), and *scrollytelling*^29^ is used to facilitate the navigation through text, images, and charts.

### 3.5. Data visualization and feedback sessions

The visualizations presented in section 4 have been co-created by the two main participants, integrating the feedback from the other participants. The work has been developed over more than 2 years of the project, during monthly work sessions. The different types of sessions were not initially defined but were designed during the monthly meetings as the project progressed. We have identified retrospectively 5 types of sessions that took place during the project and have named them to facilitate their discussion and future application.

- *Individual and collective preparation sessions*, where the participants reviewed the data available to date and validated the initial research questions (or reformulated them based on available information). Major advances in the data collection process were discussed in a meeting with the entire team, followed by individual preparation sessions and a final group session to agree on the data and the variables to be used and, in some cases, to reformulate the research questions.
- *Data visualization toolbox discovery sessions*: The data visualization researcher introduced the less-known concepts and techniques during these sessions. Additionally, possible improvements or alternatives were discussed when the *session* was used to review visualizations already created during the *sketching* and *pilot sessions* (e.g., the use of statistical graphs).
- *Sketching sessions*: During these sessions, both participants experimented with different visualization ideas, using R, Python, Excel, or Tableau for more standard charts, and paper and markers to design tailor-made visual vocabularies. Miro was also used for concept maps and brainstorming.
- *Pilot session*: In these sessions, the selected ideas were refined and completed. The result was the data visualizations that were ready to share with the rest of the team. The tools used remain the same as for standard charts, and Adobe Illustrator was used for static visualizations with custom visual vocabularies.
- *Feedback sessions*: Feedback from the rest of the team was collected during these sessions. These exchanges also brought to light the friction between the different disciplines, as we will discuss in section 5.

All these sessions contributed to the creation and application of our *data visualization toolbox*. The types of data available and the research questions that were developed during the *individual and collective preparation sessions* were the basis for the search for suitable visualization techniques. Once these techniques had been collected by the researcher in data visualization, the *data visualization toolbox discovery sessions* allowed the presentation and discussion of these techniques with other researchers. During the *sketching* and *pilot* sessions, the selected tools were applied in a practical way to the project. Finally, the results were discussed during the *feedback sessions* with the entire team. These steps were part of an iterative process that moved back and forth as new data became available, new techniques were added to the toolbox, or new feedback was received.

## 4. Results

### 4.1. Working in an interdisciplinary team

Working as an interdisciplinary team and learning how to exchange ideas, data, and experiences were at the core of this research. In this section, we discuss how, with the support of data visualization, we laid the foundations for dialog and defined the main themes of the project and the role of the different disciplines. We, therefore, focus in this section on research *question #1: How could we map knowledge and knowledge sharing to define the project scope?*

Our first interdisciplinary challenge was to map the knowledge in the team to understand the skills of each team member and how they could contribute to the project. The initial team consisted of three PhD students, three supervisors, and five supporting researchers from the different research centers involved. The foundation of any interdisciplinary project is the interest in understanding other disciplines and finding out what level of learning and collaboration is required to produce new “joint knowledge” [*trading zone* concept, see (Kemman, 2021)].

**Figure 1:**
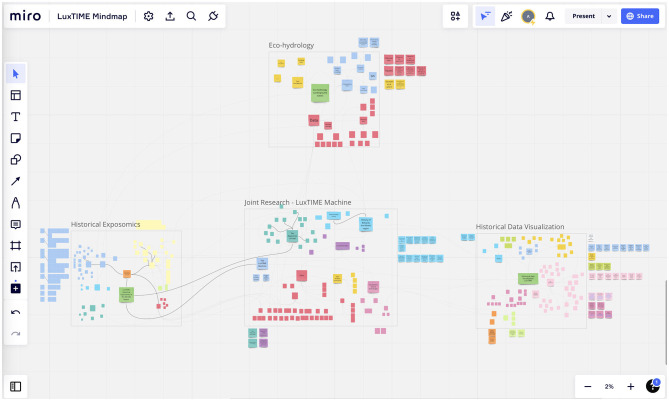
LuxTIME project concept map developed using the Miro online visual collaboration platform.

To support this process, we used a *concept map*, where we mapped the knowledge of the different disciplines and the “joint knowledge” and how they related to each other. The concept map was used to add literature about the different topics, as a starting point for the other participants to familiarize themselves with the topics, and to collect the references to the first datasets. This visualization also served as both an internal and external communication tool. The interactive online platform Miro^30^ was used for this purpose. Figure 1 shows an example of this collaborative platform, which was also used in interactive workshops such as the *UniTalks LuxTime Machine: Back to the future*, where the participants (e.g., researchers from other areas, librarians, and other university stakeholders) were encouraged to complete the concept map by adding terms and links, after reading a list of guiding questions such as “what exposures would you consider understanding the *exposome* of the population in the Minett region over the last 200 years?” and “where would you look for information?”. The result of the workshop was an enriched version of the concept map that helped us to identify new ideas and points of view.

**Figure 2:**
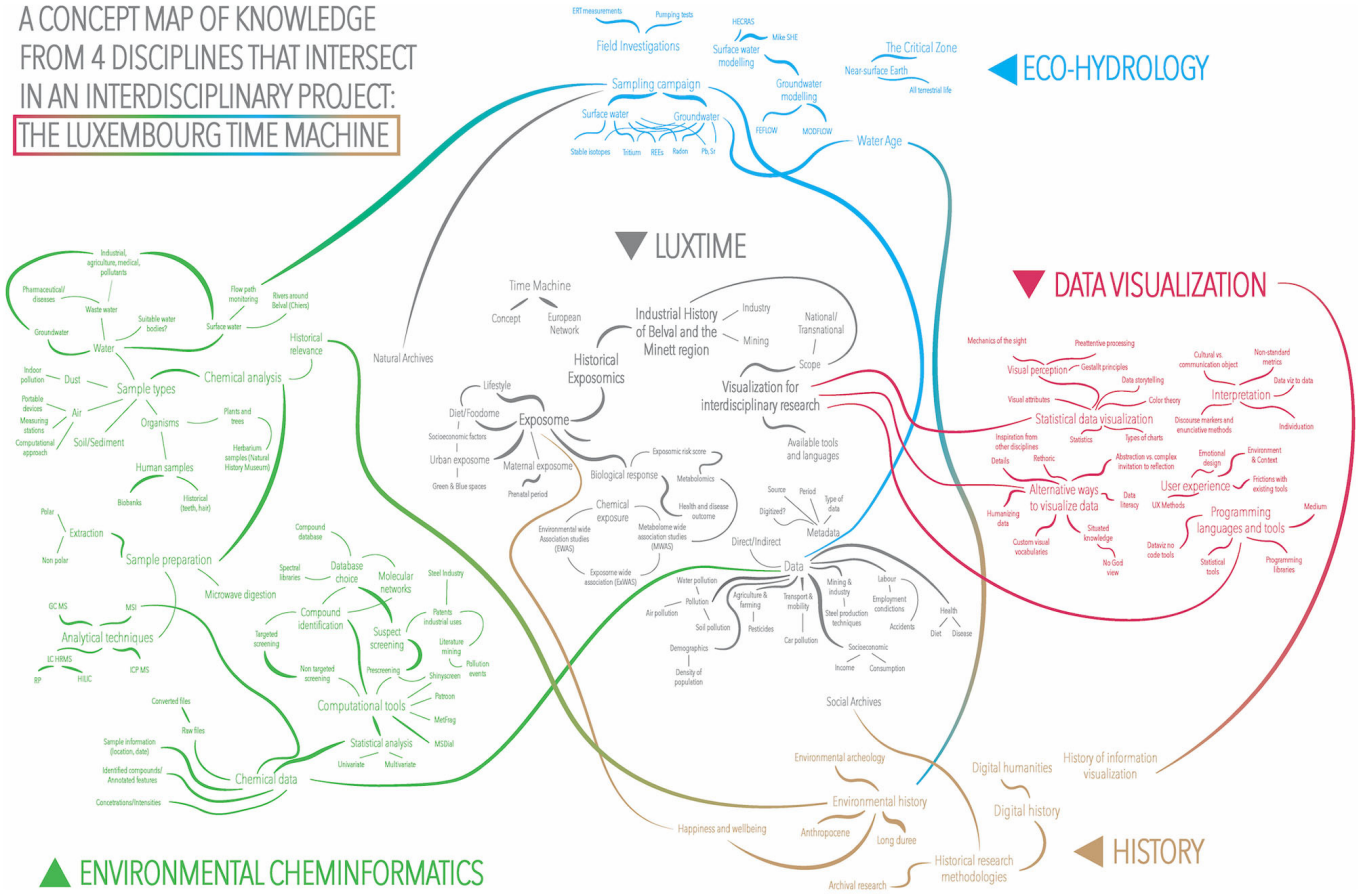
Final LuxTIME project concept map.

The final version of the concept map is shown in Figure 2. In the initial versions, we only mapped three disciplines: history, environmental cheminformatics, and eco-hydrology, displayed in different colors; and the interdisciplinary project “overlap”, in gray. In later versions, we decided to map *data visualization* as a fourth discipline, since it is not only a tool that helps to achieve a technical task but a branch of knowledge that also contributes at a theoretical level. Certain topics moved from outside “specific knowledge” to inside “joint knowledge”, such as “the exposome”, which originated in the domain of environmental cheminformatics but became the central focus of the project; or the industrial history of the Minett region, which initiated exclusively under the expertise of the historians and evolved to become a major area of joint knowledge.

### 4.2. Analyzing the process: topics, information available, and participation

As discussed above, the project involves several disciplines, with a broad area of joint overlap. Once the contributions of the participants were understood, the project scope was defined and revised throughout the project. In this section, we reflected on the process: which topics were the most relevant at each moment and why, which disciplines participated in different topics, which topics had sufficient information for further development, and which ones fell out of the project scope. Thus, here, we focus on research *question #2: How did the project evolve in terms of scope, relevance of the topics, available information, and blending of the different disciplines in each of these topics?*

The concept map allowed us to see independent snapshots of the topics at different points in time in the project, but it did not allow us to compare their evolution throughout the project in the same view, in terms of the appearance and disappearance of certain subjects, to what extent they had been integrated into the different disciplines, how relevant they were considered at each time, and what were the reasons behind these changes. The data to do this analysis were not collected during the project in a structured way, since, in this case, we wanted to use the visualization directly as a reflection tool (which could generate data later, if necessary).

**Figure 3:**
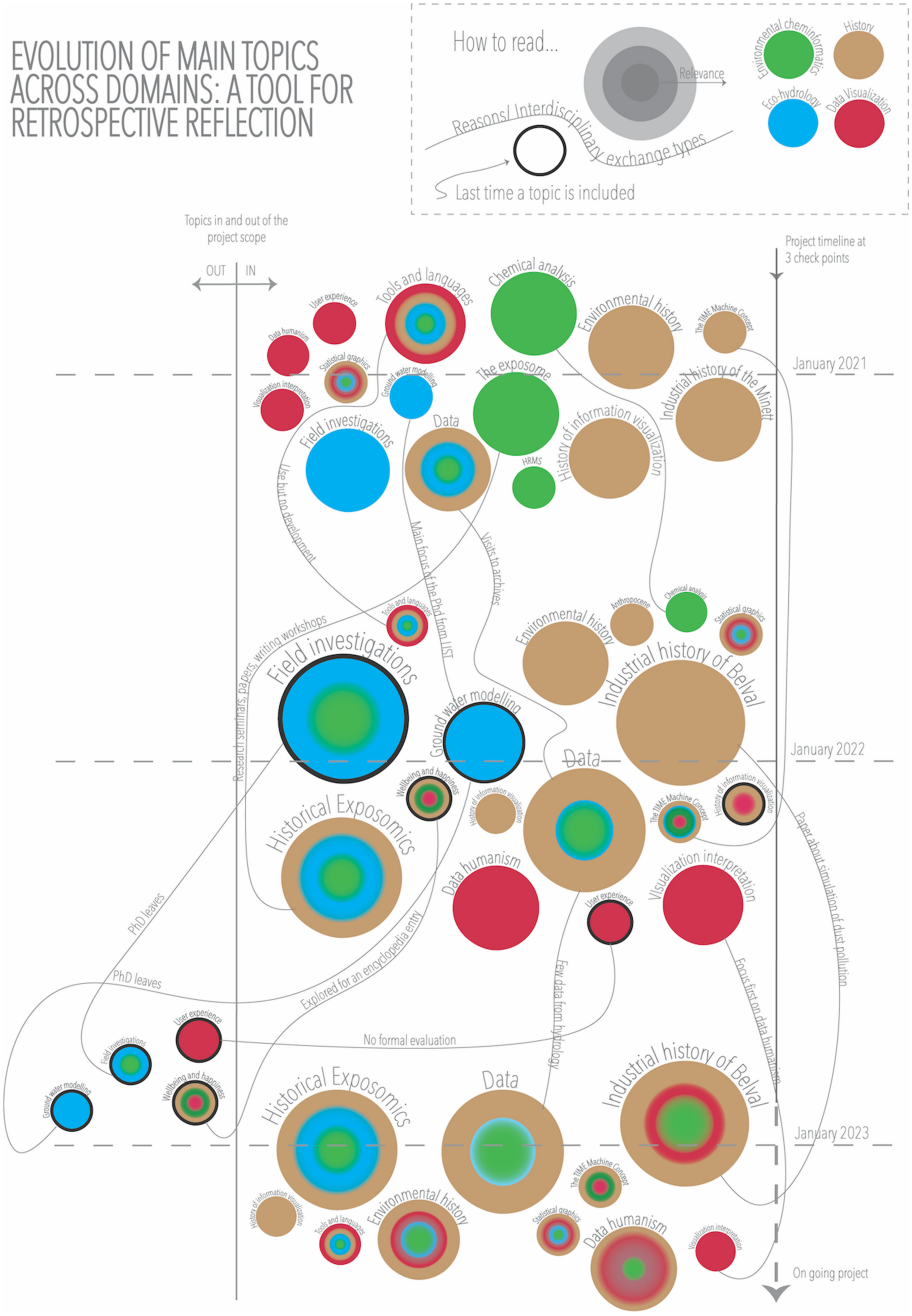
Evolution of main topics of LuxTIME.

The result of this exercise was the visualization in Figure 3, where we created a timeline with three checkpoints (January 2021, January 2022, and January 2023), around which we randomized the themes. We included a separation line “inside/outside” of the project, which allowed us to visualize which topics and around what time had been excluded. Each circle represents a topic, and the size (3 levels) indicates how important it was considered at a certain point of time in the project. The circles have a black border at the last check point before being excluded from the project scope. The color represents discipline (this color is kept consistent throughout the different visualizations in which the discipline variable is displayed). In addition, we added annotations on the lines linking topics over time to explain how a topic is integrated into different disciplines, why a topic is excluded, or how it is merged into a different topic.

In the visualization, we observed that at the beginning of the project, there were many different discipline-specific topics with lower relevance, except for the data and the tools that were common to all disciplines from the beginning. In the second checkpoint, topics such as the field investigation integrated both eco-hydrology and environmental cheminformatics with increased relevance, but then, it was excluded from the scope due to changes in the team. We also saw how the concept of historical exposomics became more important, integrating the historical and hydrological perspectives, thanks to the research seminars, literature review, and writing workshops.^31^ At the last checkpoint, only a few topics remained, with higher relevance and a blend of history, environmental cheminformatics, eco-hydrology, and data visualization. Overall, we saw a prioritization of topics, knowledge transfer, and shifting contributions among the project members, representing the functioning interdisciplinarity aspect of the project.

This visualization applied the principles of a non-representative approaches are explained in section 3.4.7, where data are not collected before the visualization is created. We used graphical features such as shapes (e.g., circles and lines), size, color, and annotations; to reflect on the evolution of the project. We drew a line connecting circles when we noticed a connection and increased or decreased the size of a circle after discussing the relative relevance of a topic at a given point in time. The existence of these elements can be registered as data, but such data did not exist beforehand, and the exercise started with the interpretative process.

**Figure 4:**
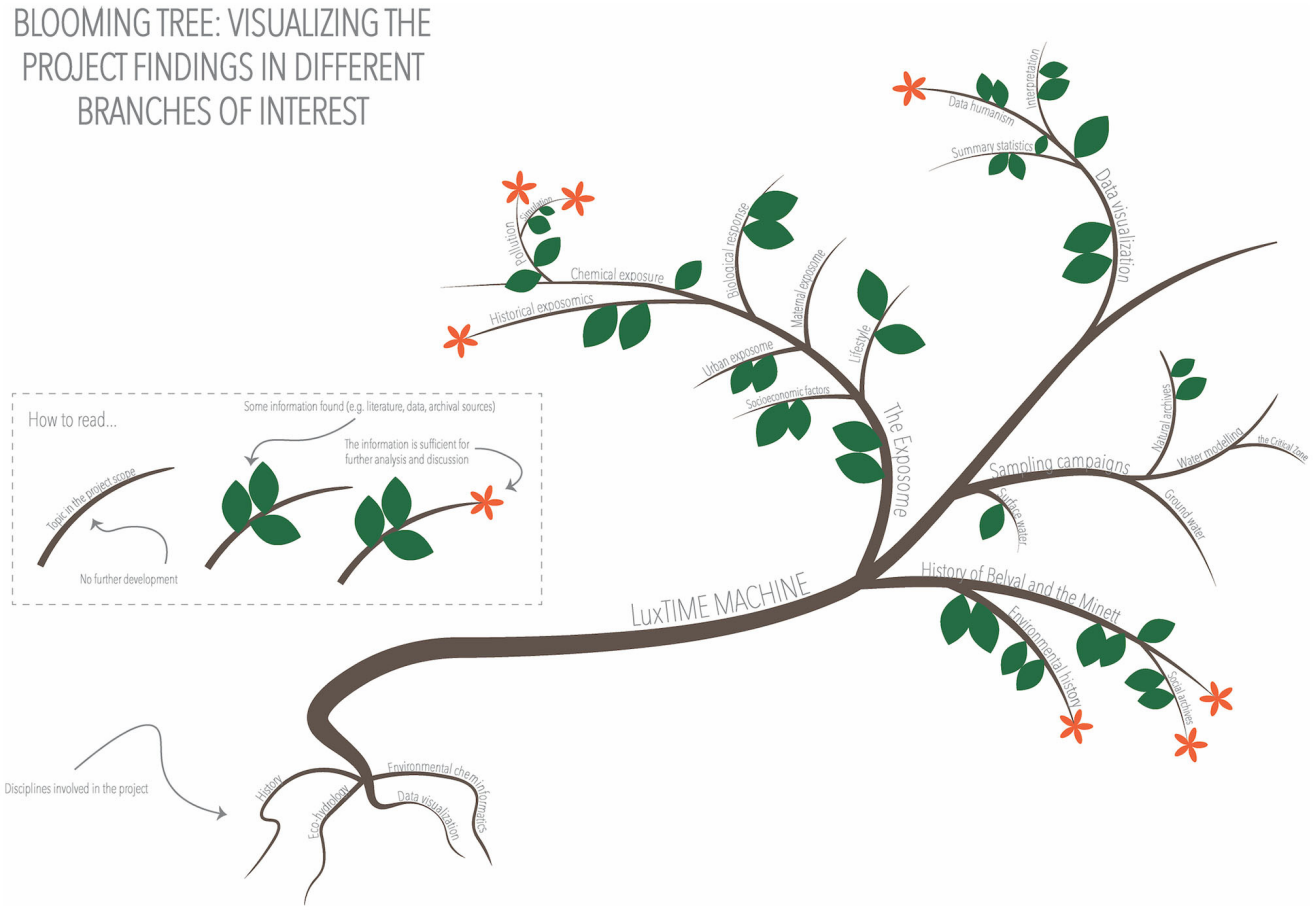
LuxTIME tree visualizing the project developments.

After defining the topics of interest, we collected all the information found through public entities, research centers, libraries, historical archives, and other sources presented in section 3.1. In the visualization in Figure 4, the objective was to display the areas with the most potential based on the information found, i.e., the “flourishing branches” (see visual rhetoric is described in section 3.4.4 at a given moment). We defined three levels: The first was just the branch, which stated that the topic had been considered and researched; the second level was illustrated with a leafy branch, indicating that some information had been found (e.g., historical sources mentioning the topic, existing previous research, and literature available); and the third one, a flowering branch, depicted the possibility of further analysis to extend the research (e.g., data can be extrapolated to the Minett region, it concerns the period of interest, or a projection can be done). The tree, at the same time, allowed us to separate the branches into themes and sub-themes and to represent the roots of the project, the four disciplines. As we can see in Figure 4, the most developed branches, at the time of visualization, were pollution, data humanism, historical exposomics, environmental history, and the discovery of social archives about the history of the Minett region. Other topics for which some information had been found but was not yet sufficient for further analysis included other areas of the *exposome* (e.g., urban exposome, lifestyle, and biological responses) or the sampling campaigns. This visualization could be annotated to explain why these areas had been further developed, which types of data had been found, where, and what analyses they allowed. It could also be combined with a second visualization showing the details, an animation (e.g., showing a tree whose leaves appear, the flowers bloom and then wilt, and the leaves fall off), or adding interactivity that has been neglected in this first visualization phase.

The use of the tree allowed us to use multiple visual metaphors, the roots representing the disciplines, support, and nourish the project; the branches grow and branch out as the project progresses, and the leaves and flowers bloom due to multiple factors. This visualization showed the progress of the project without using any conventional numerical or graphical charts and could be used to communicate with all kinds of audiences. It also showed which branches of the project were “blooming” at first glance, as a starting point to discuss how to move forward.

### 4.3. Exploring data and metadata

One of the most challenging aspects of the project was the data collection. In addition to the complexity due to the wide range of topics, there were also different types of sources (e.g., texts, images, and maps) and archives. The inventory of the information continues, as well as the analysis of the different datasets. This section focuses on research question #3 using selected examples: *How could we explore the data and metadata respecting the priorities of the different disciplines?*

#### 4.3.1. Visualizing metadata of the information collected

**Figure 5:**
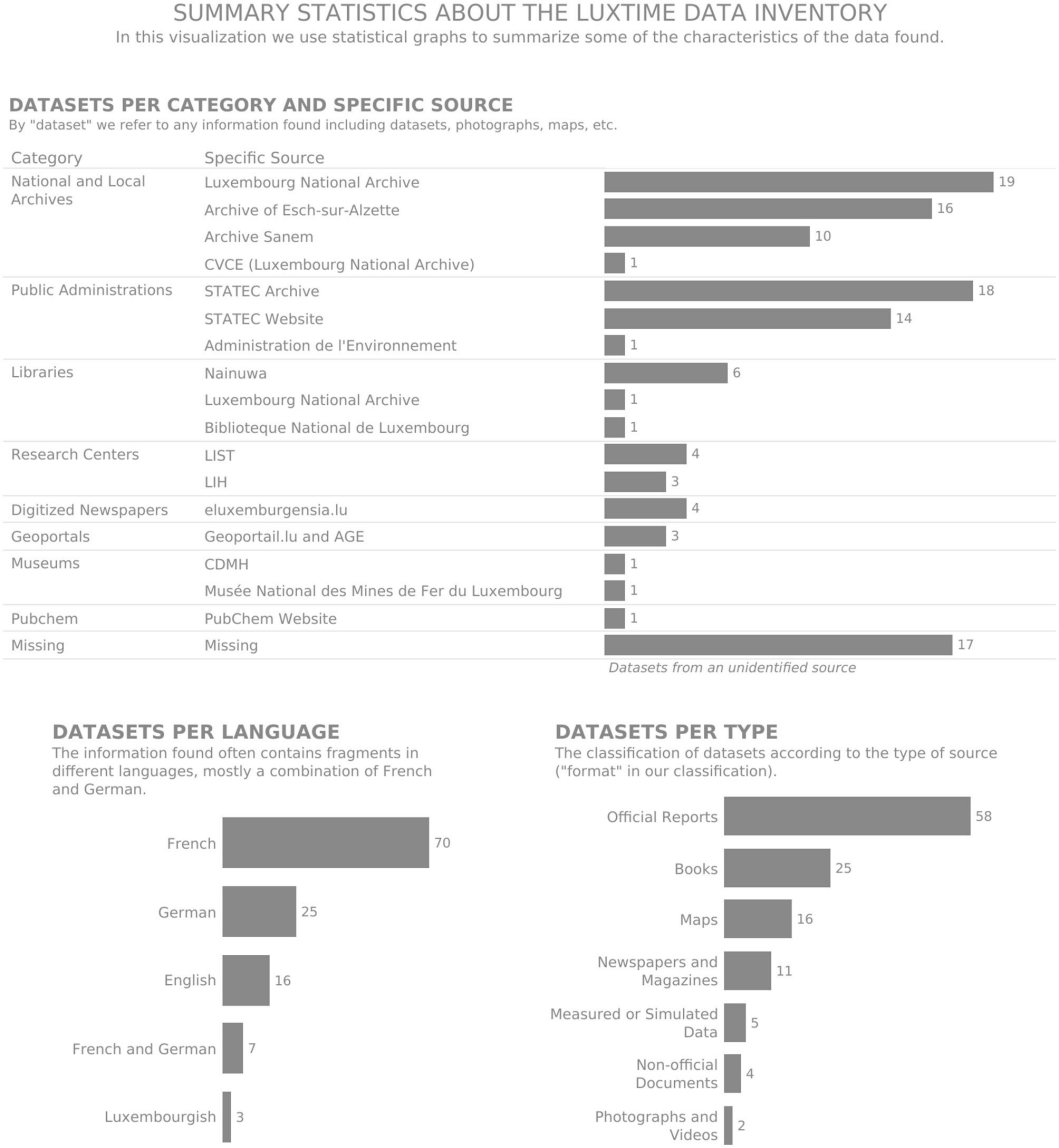
Summary statistics about the LuxTIME data inventory.

The use of statistical graphs, such as bar charts allowed us to explore individual variables of the inventory, answering questions such as *How many datasets per source did we have?* This question could be easily answered using summary statistics (Figure 5), as the inventory contains 21 datasets from the Luxembourg National Archive, 18 from STATEC, 16 from the Archive of Esch-sur-Alzette, etc.

**Figure 6:**
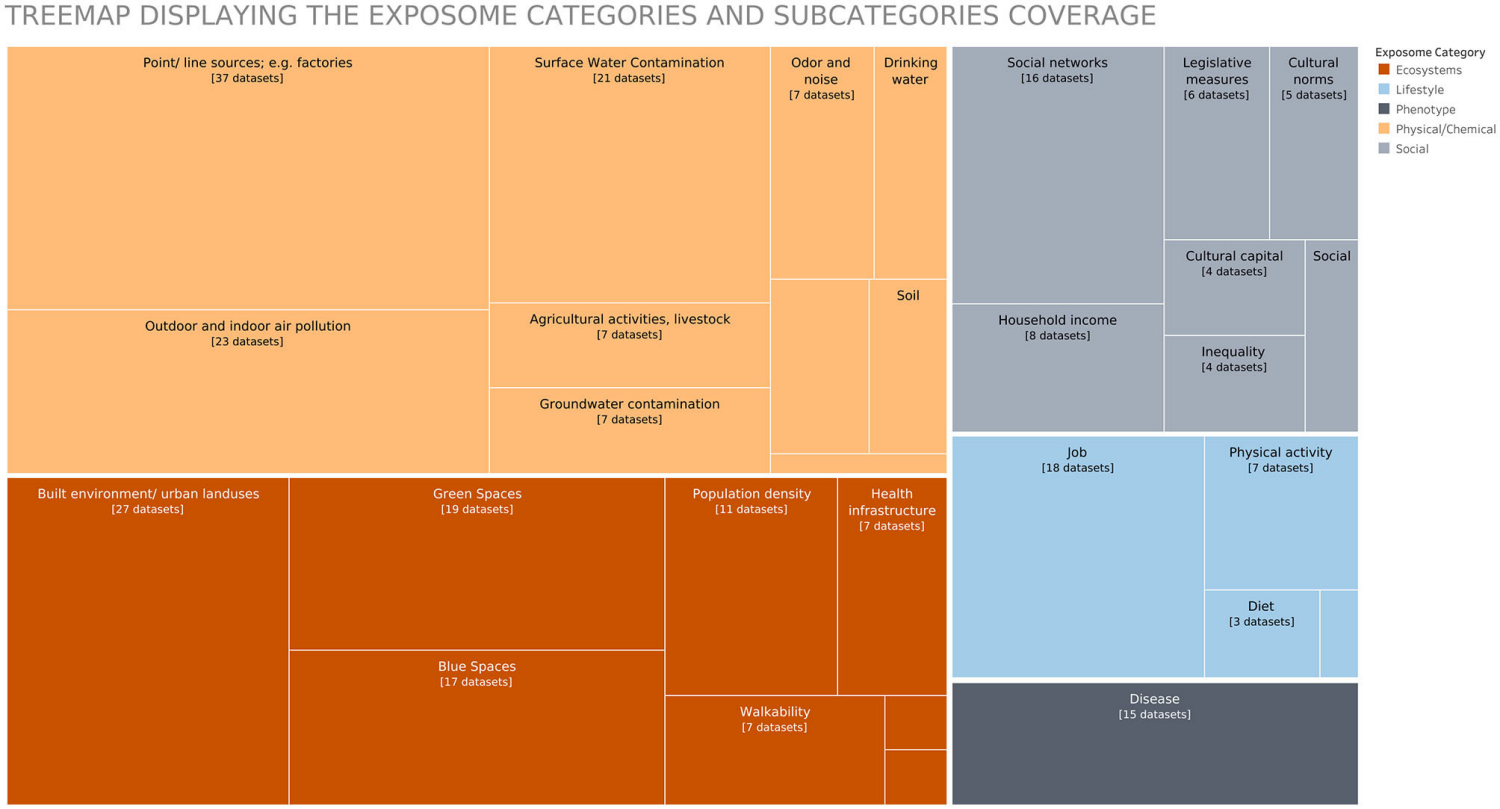
Treemap displaying the data inventory by exposome category and subcategory.

*How many datasets covered every category and subcategory of the exposome?* We did a subdivision of the *exposome* into five categories (Ecosystems, Lifestyle, Social, Physical/Chemical, and Phenotype) and 43 subcategories. In Figure 6, we visualized this using a treemap, with the number of datasets related to the different categories and subcategories. Most datasets belonged to several classification categories, which are not displayed in the treemap. The analysis of this overlap would require a different visualization. In the graph, we could see that the categories with the most information were physical/chemical and ecosystems, followed by social and lifestyle.

**Figure 7:**
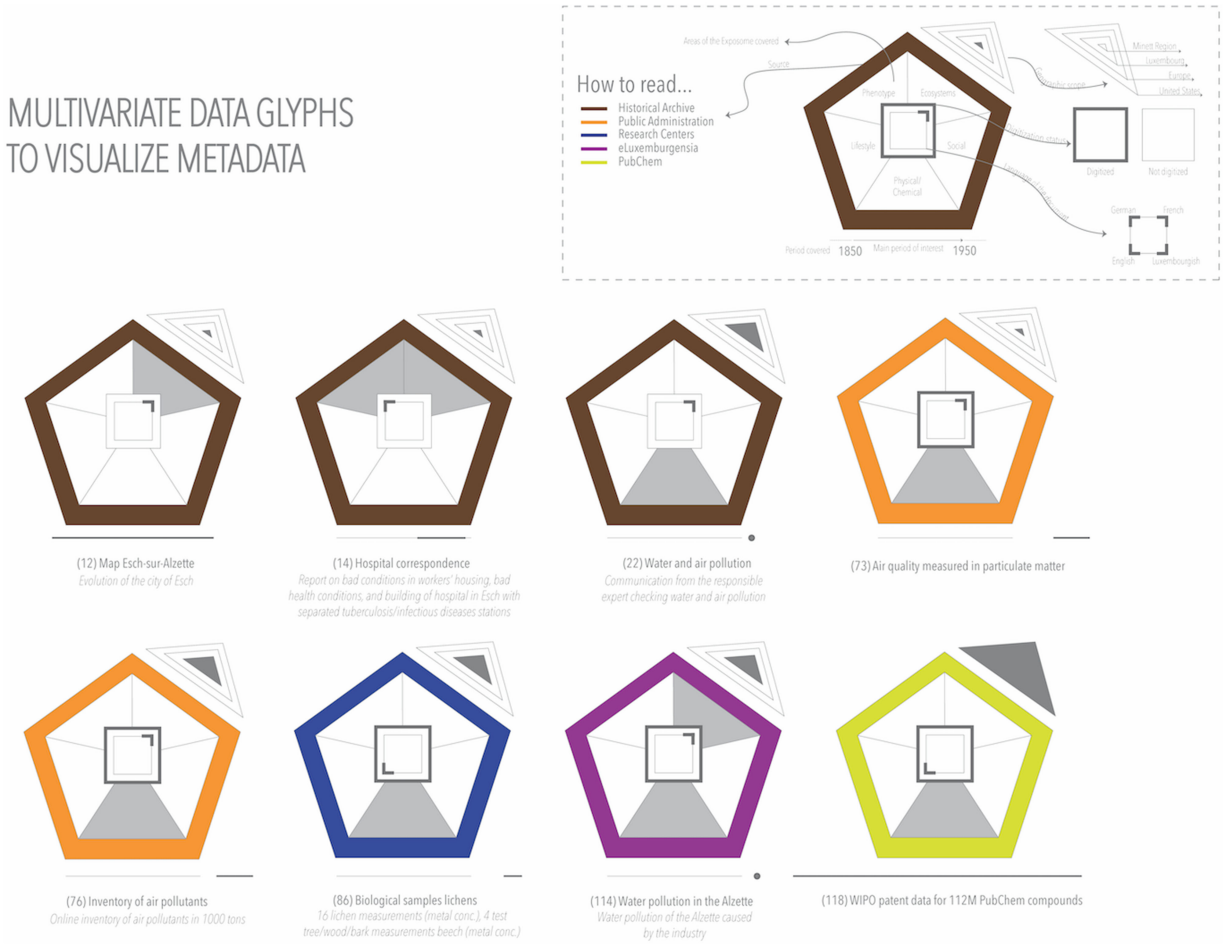
Multivariate data glyphs.

The visualizations in Figures 5, 6 allowed us to summarize the metadata, an analysis that could be extended to all the collected metadata in the inventory (e.g., time period, geography, and authors). However, we could not include multiple variables in the same view and still see the details of each of the collected datasets. For this purpose, we used multivariate data glyphs (see Figure 7). One of the principles of *data humanism* (Lupi, 2017) already introduced in section 3.4.5 is the use of dense and unconventional data visualizations to promote exploration, as it requires the reader to become familiar with the visual encoding, and it layers multiple visual narratives for the readers to follow their own interest “since clarity does not need to come all at once”.

As we see in Figure 7, we selected a series of graphical attributes customized to the visualization. The color of the outer pentagon represents the source. In the inventory, we collected 121 datasets from 17 sources, which we grouped for visualization into national and local archives, museums, libraries, research centers, the geoportal, public administrations, and eluxemburgensia. The inner pentagon, in gray, represents the areas of the *exposome* covered in the dataset. The outer square with a thick gray border indicates that the source has been digitized, and each corner of the inner square indicates in which language it is available. At the bottom of the pentagon, the period covered by the data was indicated, with the base coinciding with the period of interest of the project, between 1,850 and 1,950. The concentric triangles at the top right indicate from the inside out the geographical area covered by the data, from the Minett (smallest triangle) to a global scope (largest). This visualization, despite requiring more time to explore, would allow us to see different aspects at the same time, without losing sight of the granularity of the dataset. Further iterations of glyphs and layouts will be evaluated before final implementation.^32^

#### 4.3.2. Exploring a dataset: number of chemicals registered over time

After having explored the metadata of the data inventory, we started to analyze the datasets. Given the variety of datasets in the inventory, each required a particular analysis to define the required data visualizations. We have chosen, as an example, a set of data about the number of chemicals registered in the Chemical Abstracts Service (CAS) registry^33^ over time.

**Figure 8:**
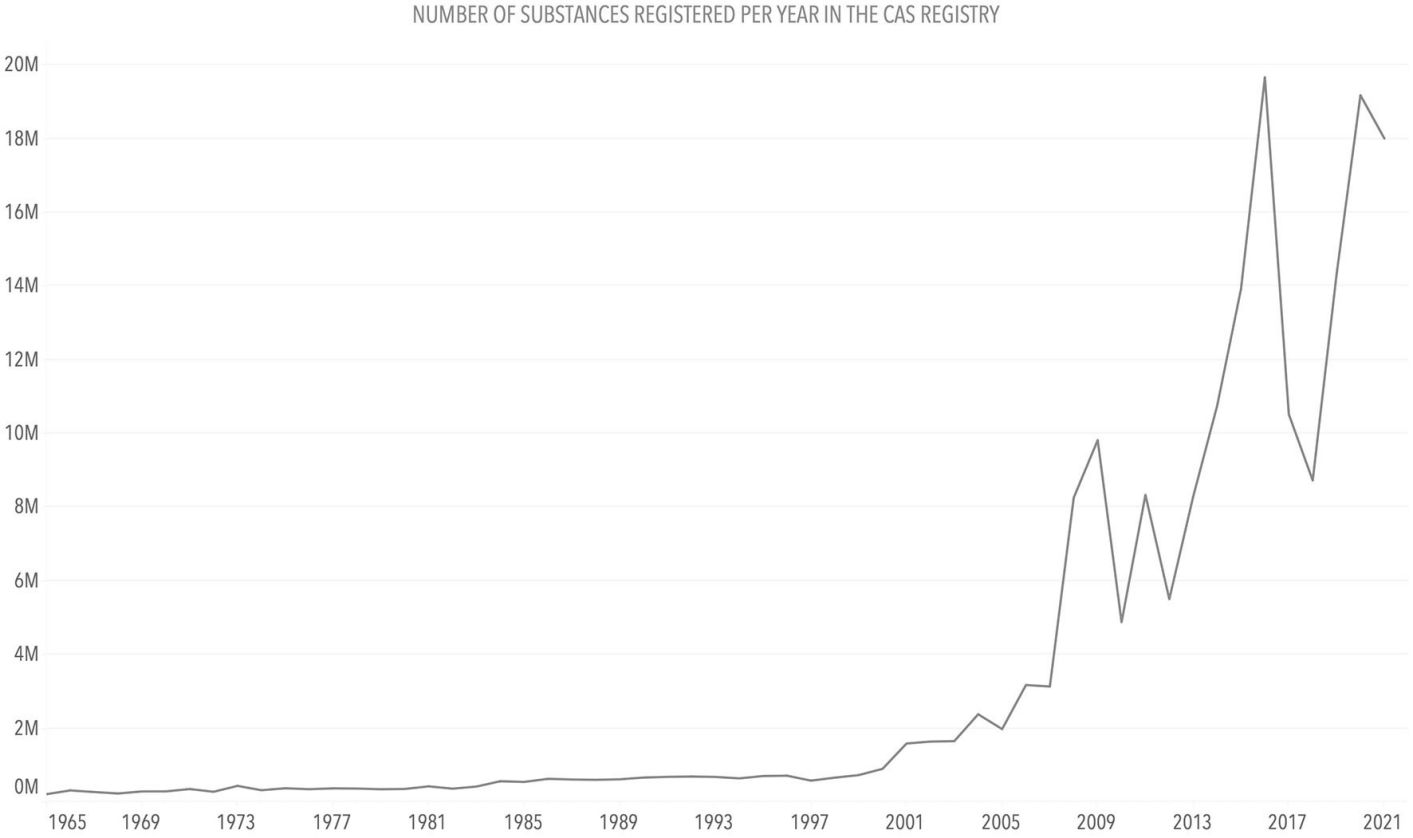
Line chart: Number of unique substances registered in the CAS registry per year. Source: CAS registry.

**Figure 9:**
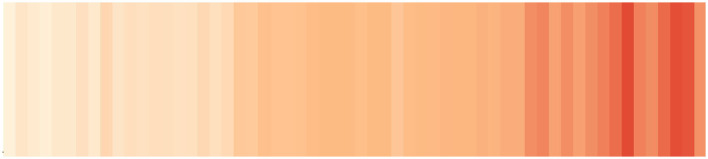
Stripes: Number of unique substances registered in the CAS Registry per year. Source: CAS registry.

Figure 8 shows a classical representation of quantitative (chemical) data using a line chart. As an alternative, in Figure 9, *chemical stripes* (Arp et al., 2023) (inspired by the *warming* or *climate stripes* discussed in section 3.4.2) are shown, presenting trends of chemical registrations in the CAS registry since 1965, with low numbers in light red and high numbers in dark red. In Figure 9, the color hue was chosen to alert about the situation of increasing chemical numbers, while the color values (from light to dark) allowed the use of red and made it possible for readers with color deficiencies to see the differences.

The numbers of chemicals registered in the CAS registry do not correspond directly to the number or amount of chemicals in use. The stripes could be created for different compound classes or a group of several chemicals, having the registration numbers for these individual requests. The general trend remains the same, irrespective of the view or data: The number of chemicals in use and present in the environment increases along with the number of chemicals. The total number of registered unique substances in the CAS registry lies over 200 million substances currently, with 10–20 million new entries added per year. As in the case of the *climate stripes*, this simplified heatmap conveyed a clear message about the increasing number of chemicals. As discussed in section 3.4.2, this is an example of a variation of a statistical graph, a heatmap, where the graph elements (e.g., axes, legend, and tick marks) are removed to draw attention to a single message, the increasing or decreasing values through the color hue. The objective is not to provide exact values to the reader but to show a noticeably clear trend through minimalism in the visualization.

### 4.4. Monitoring project deliverables and experience

Publications are a fundamental part of the results of a research project, including the various parts of the publication process and the experience of the participants. However, the analysis of this process was not only to optimize the logistics of the project but to embrace a hermeneutic analysis based on interpretation, to understand how the publication processes differ among disciplines, what kind of publications predominate and why, and how it changes in more or less interdisciplinary, individual, or shared publications. We used data visualization not only for the purpose of quantitative analysis (e.g., how many papers had been published) but as a tool for close reading, to develop a deeper understanding of the process itself. In this last section of the results, we focus on *research question #4: How could we monitor the project deliverables, including the different steps of the process, the contribution of the different disciplines, and the experience of the participants?*

In previous examples, we have discussed two time-based visualizations: the evolution of the project scope around three checkpoints throughout our project (Figure 3), and a simplified heatmap to visualize the change of a variable (e.g., CAS registration numbers; Figure 9) over time. Next, we were interested in monitoring the progress of planned publications during the project. We wanted to know the start date of the work, the date of publication, and also the intermediate steps (e.g., when it is submitted and the progress toward acceptance). We also wanted to know which disciplines participated in each publication, whether authors from outside the project team were involved, and what type of publication it was (e.g., article, poster). The visualization technique most frequently used to represent time intervals is Gantt charts. However, a simple Gantt chart did not allow us to represent all the variables of interest in this analysis.

**Figure 10:**
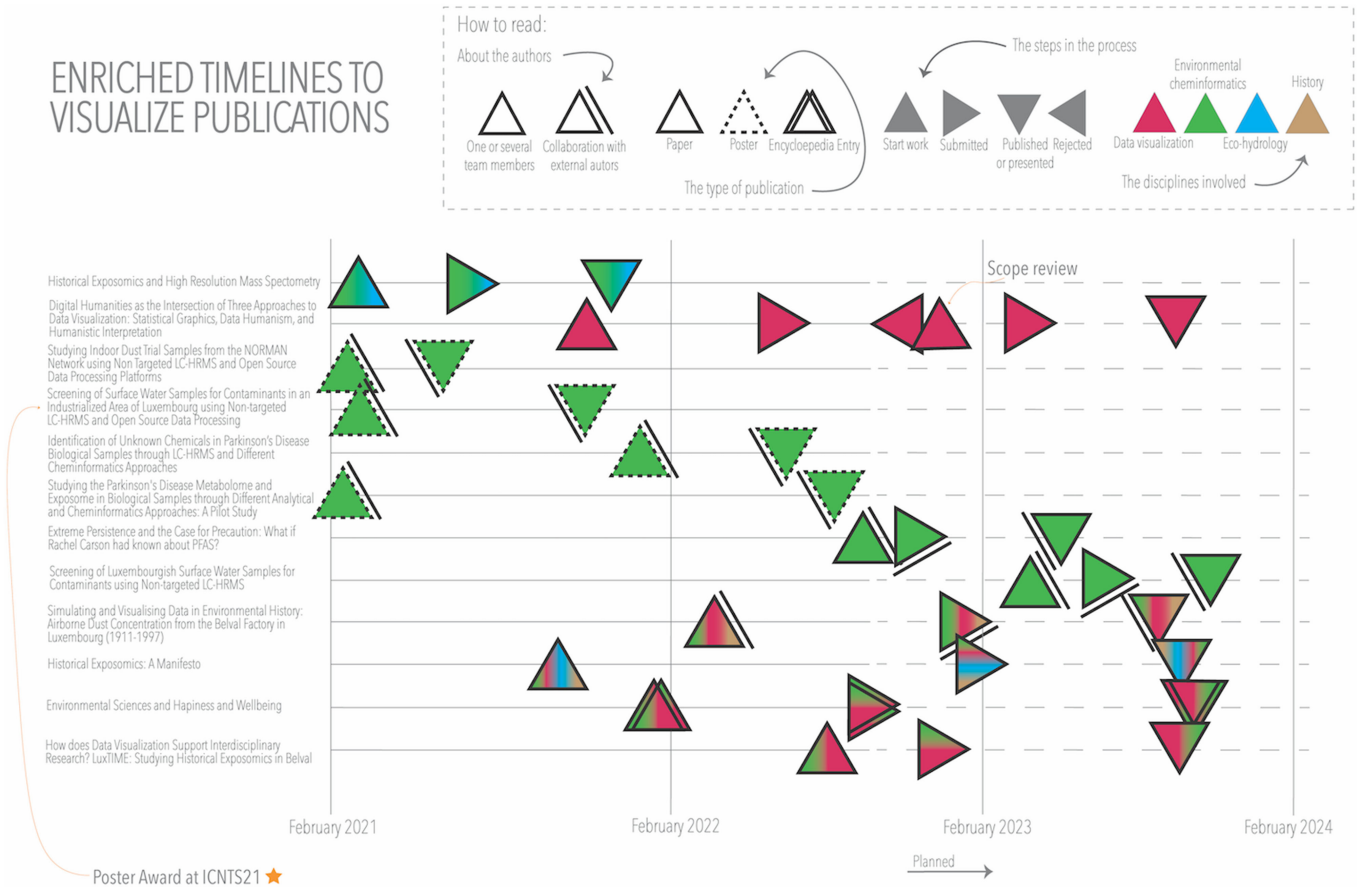
Team publications.

In Figure 10, we can see how we could enrich the information shown in a temporal diagram. By combining multivariate data glyphs and placing them at specific points on the timeline, we could divide the process into different steps between the time of starting the work and the publication. We displayed a timeline for each publication, with a triangle at each stage of the process, rotating to the right when the process moves forward (e.g., submission), and to the left when it “goes backwards” (e.g., rejection/revision leading to a restart of the submission process). The color of the triangles indicates that there are individual publications for each discipline, as well as combinations of two or more disciplines. Publication types for environmental cheminformatics (green) include three posters (dashed triangles), seven articles, and an encyclopedia entry. We can also see that in five publications, authors from outside the project are involved. This visualization allows us to see at which moments work accumulates and why. The rotation effect of the triangle helped us to understand parts of the process, for example, the effect of “going backwards” even as time moved forward, due to a rejection that required a restart. At the same time, the rotation of the triangle prevented us from seeing when the event occurred with precision (i.e., vertex or center), but as in this visualization, we did not need exact dates; we chose to keep the triangular shape and take advantage of the rotation effect.

**Figure 11:**
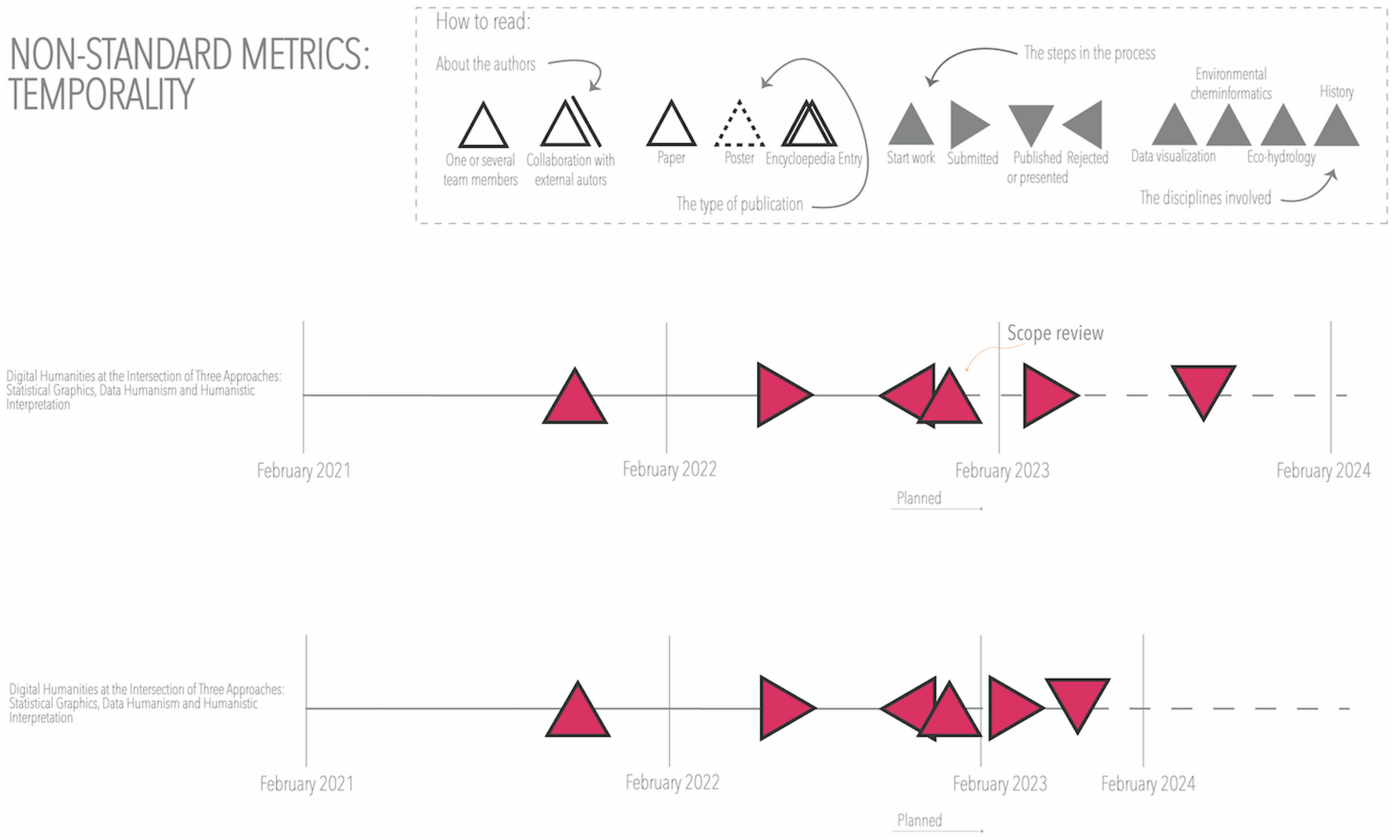
Non-standard time intervals.

The time scale could be visualized in more detail (e.g., with monthly details), adding more steps to the process (e.g., differentiate acceptance and publication), or factoring in the time perception of different participants, using non-standard intervals. For example, one of the authors might have perceived the initial time spent working on a publication differently from the time spent working after the rejection of the initial version (see Figure 11). The visualization in Figure 11 highlights the difference between time and temporality, the latter being relational. It opens the visualization techniques to graphical methods that represent experiential temporality, a subjective experience that depends on many psychological and physiological factors. If different participants of the project were to repeat the visualization for different publications, the challenges related to the different points of view of the multiple sources would need to be accounted for as discursive temporality. The visualization of time—based on experience—allows us to explore how the different participants experience the project (e.g., which moments are perceived as most stressful). This visualization technique, where the timeline is not standard, is probably the one that takes epistemological differences the furthest.

## 5. Discussion

In this article, we have presented the LuxTIME *data visualization toolbox*, including several data visualization concepts and techniques from epistemologically distant disciplines. This *toolbox* has facilitated an interdisciplinary collaboration among researchers in history, environmental cheminformatics, eco-hydrology, and data visualization, learning and applying concepts and techniques that reflect the paradigms of the other disciplines (e.g., integrating the use of rhetoric or temporality in natural sciences). Through the data collection process, the numerous exchanges on data visualization, the sketching and prototype development sessions, and the feedback collection and implementation, we have experienced and learned about the frictions inherent in interdisciplinary work.

First, the different perspectives on the level of granularity are worth noting, a friction already described by Panagiotidou et al. (2022). Especially, in the visualization process, we had several discussions about how important the details were in the visualizations (i.e., how we reached the results through several iterations with positive and challenging experiences: failure, team changes, and learning opportunities) vs. just visualizing the result. Second, by integrating the ideas of *data humanism* and *interpretation* into our *toolbox*, we increased the time needed to explore some of the visualizations, which often triggered the “discomfort” of not being able to immediately arrive to a clear conclusion. For example, in multivariate visualizations, there are several levels of information that cannot be extracted at first glance, but they allow us to show many facets of the metadata in a single view if we are willing to spend more time in the exploration. Third, the most accepted concepts were the integration of rhetorical mapping, and the variations of statistical graphics, notably the minimalist approach (i.e., no axes, tick marks, or grids), probably because it already had a strong precedent with the climate stripes.

Finally, one of the elements that we added to the *toolbox* at a later stage, because the need emerged, was the correct use of statistical graphics. Although statistical graphics were often used in all the disciplines involved, the visualizations generated did not always respect established theories on data visualization research. These discussions (e.g., color theory) highlighted the need to collaborate with researchers in data visualization so as not to perpetuate errors within the disciplines. Moreover, the fact of including concepts and techniques that serve different objectives (e.g., drawing quick conclusions using perception theory vs. exploring in-depth multiple narratives through multivariate visualizations with custom visual vocabularies) in our *toolbox*, created a series of discussions about how blurred the lines are between such “tools” and the paradigms they come from.

Throughout the research, the idea of data visualization as a *sandcastle* has been very present, especially the use of data visualization as part of the *speculative process*, and not just to present the results once the work is done (Hinrichs et al., 2019). This research is an attempt to use data visualization not only to explore and communicate about the domain data and metadata but also to collect and explore forms of thinking and creating knowledge through visual means. To make this visual means more specific, we have collected a series of existing concepts and techniques, our *toolbox*, to encourage participants to rethink what they already know but also to defamiliarize themselves with the usual methods and use data visualization as an *aesthetic provocation* (Hinrichs et al., 2019) to open up new perspectives for their own disciplines and the interdisciplinary work at LuxTIME. Throughout the process, visualization in its role as a *mediator* promotes an open and critical discourse (Hinrichs et al., 2019).

All the visualization concepts and techniques discussed in this article are just a proposal for a *data visualization toolbox* suitable for many research fields. Such concepts and techniques are not new but are rarely combined across disciplines or within the framework of a single project. In LuxTIME, we wanted to experiment with different data visualization techniques in a practical way, through our own process of interdisciplinary learning and exchange, during the definition of the project, and to explore the data and metadata found in relation to our main theme: historical exposomics in the Minett region. The aim was to extend the “go-to” *data visualization toolbox* and to explore, validate, and communicate, benefiting from techniques researched and applied across different disciplines. The use of a variety of techniques allowed us to look at data from different perspectives, analyzing the process quantitatively, qualitatively, and in an interpretative manner (e.g., combining Gantt charts with more flexible and detailed views, using non-standard timelines to express the experience of participants), and alternating and combining the use of statistical graphs with the use of metaphors or other graphic elements, whose aim is not necessarily to communicate quickly and accurately but to foster emotions.

This combination of statistical graphics and their variations, the use of information design elements generally present in the so-called *data art*, as well as the integration of interpretative elements gave us an *extended data visualization toolbox* to navigate our project. Probably because of the interdisciplinary nature of the project, this combination was more obvious, but we believe it could be useful for projects of all kinds. Such a *playground* is necessary to be able to formally evaluate the combined use of these techniques in future. This exploratory practice refers to the notion of “thinkering”, composed of the verbs tinkering and thinking that describes the action of playful experimentation with digital tools for the interpretation and presentation of history (Fickers and van der Heijden, 2020). There is no one-size-fits-all toolbox for every research project, as the toolbox concept is built on the idea of flexible rearrangements of tools depending on the research questions, needs, and aims of a project. The toolbox is therefore the result of a “co-design” process based on situated knowledge practices.

The visualizations presented in this article are prototypes that will evolve further toward the end of the project, incorporating other techniques (e.g., interactivity, direct visualization, and enhanced ways of storytelling). Moreover, after having experimented with several types of visualizations separately to address different research questions, future steps will include a review of the connections between different visualizations and how they are linked in the overall narrative.

## Data availability statement

The data analyzed in this study is subject to the following licenses/restrictions: Data will be published at the end of the project in 2024. In this article we just discuss the data visualizations. Requests to access these datasets should be directed to aida.horanietibanez@uni.lu and dagny.aurich@uni.lu.

## Ethics statement

Ethical approval was not required for the study involving human participants in accordance with the local legislation and institutional requirements. Written informed consent to participate in this study was not required from the participants in accordance with the national legislation and the institutional requirements.

## Author contributions

All authors participated in the conception and design of the study, performed the data collection, data analysis, data interpretation, drafting of the article, contributed to the manuscript revision, and read and approved the submitted version.

## Footnotes

^2^ https://luxtimemachine.uni.lu/

^3^ https://www.timemachine.eu/

^4^LuxTIME INITIATE aims at building a consortium with the main stakeholders of historical data in Luxembourg (archives, libraries, museums, statistical offices, research institutions, governmental bodies, and private associations) to study the past in an interdisciplinary inter-institutional setting, with both intellectual and technical impact.

^5^The theory behind the concepts and techniques mentioned below might overlap but each of them has in some way proven to be useful during our project, and therefore, we considered it worthwhile including them all in our *toolbox*.

^6^Designer refers to the person who designs the visualization (i.e., statistical charts), independent of their main area of expertise.

^7^ https://showyourstripes.info/s/globe

^8^ https://multimedia.scmp.com/china-overseas-investments/

^9^ https://fivethirtyeight.com/features/where-we-saw-red-and-blue-mirages-on-election-night/

^10^ https://fivethirtyeight.com

^11^ http://sciencepaths.kimalbrecht.com/

^12^ https://www.nationalgeographic.com/magazine/graphics/graphic-shows-past-50-years-of-global-human-migration?sf215829698=1&sf217104276=1

^13^The data-ink ratio is a concept introduced by Tufte (1999) as the proportion of ink that is used to present actual data compared to the total amount of ink (or pixels) used in the entire display.

^14^ https://www.sarahemeryclark.com/work/whats-cookin

^15^ https://emotions.periscopic.com/inauguration/

^16^ http://poppyfield.org

^17^ http://www.studioterp.nl/a-view-on-despair-a-datavisualization-project-by-studio-terp/

^18^ http://giorgialupi.com/data-items-a-fashion-landscape-at-the-museum-of-modern-art

^19^ http://giorgialupi.com/bruises-the-data-we-dont-see

^20^ http://metoomentum.com/trending.html

^21^ https://kadambarik.myportfolio.com/data-selfi

^22^ http://www.dear-data.com

^23^ https://www.5avestreettrees.com

^24^ https://www.lucasinfografia.com/Front-pages-analysis

^25^ https://www.behance.net/gallery/138862771/Women-in-politics?tracking_source=search_projects%7Cinformation%20visualization%20data%20art

^26^ https://domesticstreamers.com/projects/the-mood-test/

^27^ https://threedh.net

^28^https://www.framingluxembourg.lu, developed by the C2DH in collaboration with STATEC.

^29^*Scrollytelling*, from “scrolling” and “storytelling”, is a way to display content that unfolds as the user scrolls.

^30^ https://miro.com

^31^LuxTIME Seminar Series organized throughout the project to facilitate the exchange of knowledge and stimulate the discussions within and outside the working group: https://luxtimemachine.uni.lu/#1620717521789-01362bff-416e.

^32^All visualizations are prototypes developed throughout the project, at the end of which, a final version will be integrated into the project website https://luxtimemachine.uni.lu/.

^33^ https://www.cas.org/cas-data/cas-registr

## References

[B1] Aigner W. Miksch S. Schumann H. Tominski C. (2011). Visualization of Time-Oriented Data. London: Springer London. 10.1109/TVCG.2007.70415 17993701

[B2] Arp H. P. H. Aurich D. Schymanski E. L. Sims K. Hale S. E. (2023). Avoiding the next silent spring: our chemical past, present, and future. Environ. Sci. Technol. 57, 6355–6359. 10.1021/acs.est.3c01735 37053515 PMC10134483

[B3] Aurich D. Miles O. Schymanski E. L. (2021). historical exposomics and high resolution mass spectrometry. Exposome 1, osab007. 10.1093/exposome/osab007

[B4] Cairo A. (2019). How Charts Lie: Getting Smarter About Visual Information. 1st ed. New York, N.Y.: W.W. Norton and Company.

[B5] Chuah M. C. Eick S. G. (1998). Information rich glyphs for software management data. IEEE Comput. Graph. Appl. 18, 24–29. 10.1109/38.689658

[B6] Cleveland W. S. McGill R. (1984). Graphical perception: theory, experimentation, and application to the development of graphical methods. J. Am. Stat. Assoc. 79, 531–554. 10.1080/01621459.1984.10478080

[B7] Cleveland W. S. McGill R. (1986). An experiment in graphical perception. Int. J. Man-Mach. Stud. 25, 491–500. 10.1016/S0020-7373(86)80019-0

[B8] Cordell R. (2019). “Teaching humanistic data analysis,” in Digital Scholarship, Digital Classrooms: New International Perspectives on Research and Teaching: Proceeding of the Gale Digital Humanities Day at the British Library.

[B9] D'Ignazio C. Klein L. F. (2020). Data Feminism. Cambridge, MA: The MIT Press.

[B10] Dörk M. Feng P. Collins C. Carpendale S. (2013). “Critical InfoVis: exploring the politics of visualization” in CHI'13 Extended Abstracts on Human Factors in Computing Systems (Paris France: ACM), 2189–2198.

[B11] Drucker J. (2011). Humanities Approaches to Graphical Display. Boston, MA: DHQ. p. 5.

[B12] Drucker J. (2018). Non-representational approaches to modeling interpretation in a graphical environment. Digit. Scholarsh. Humanit. 33, 248–263. 10.1093/llc/fqx034

[B13] Drucker J. (2021). TimeCapsule Report on Research 2001-2021. UCLA, Department of Information Studies and Design.

[B14] Dykes B. (2020). Effective Data Storytelling: How to Drive Change With Data, Narrative and Visuals. Hoboken, NJ: John Wiley and Sons, Inc.

[B15] Evergreen S. D. H. (2017). Effective Data Visualization: The Right Chart for the Right Data. Los Angeles, CA: SAGE.

[B16] Fickers A. Tatarinov J. Fickers A. Tatarinov J. van der Heijden T. (2022). “Digital history and hermeneutics – between theory and practice: an introduction,” in Digital History and Hermeneutics: Between Theory and Practice (Berlin: De Gruyter).

[B17] Fickers A. van der Heijden T. (2020). Inside the Trading Zone: Thinkering in a Digital History Lab. Boston, MA: DHQ. p. 14. Available online at: http://digitalhumanities.org/dhq/vol/14/3/000472/000472.html (accessed September 05, 2023).

[B18] Glivinska A. (2021). The 25 Best Data Visualizations of 2023 [Examples]. Visme. Available at: https://visme.co/blog/best-data-visualizations/ (accessed January 13, 2023).

[B19] Hall K. Wm Bradley A. J. Hinrichs U. Huron S. Wood J. . (2020). Design by immersion: a transdisciplinary approach to problem-driven visualizations. IEEE Trans. Vis. Comput. Graph. 26, 109–118. 10.1109/TVCG.2019.2934790 31449025

[B20] Hill C. A. Helmers M. (2009). Defining Visual Rhetorics. Reprinted from Lawrence Erlbaum ed. New York, NY: Routledge.

[B21] Hinrichs U. Forlini S. Moynihan B. (2019). In defense of sandcastles: Research thinking through visualization in digital humanities. Digit. Scholarsh. Humanit. 34, i80–i99. 10.1093/llc/fqy051

[B22] Hullman J. Diakopoulos N. (2011). Visualization rhetoric: framing effects in narrative visualization. IEEE Trans. Vis. Comput. Graph. 17, 2231–2240. 10.1109/TVCG.2011.255 22034342

[B23] Karlsson O. Rocklöv J. Lehoux A. P. Bergquist J. Rutgersson A. Blunt M. J. . (2021). The human exposome and health in the Anthropocene. Int. J. Epidemiol. 50, 378–389. 10.1093/ije/dyaa231 33349868 PMC8128460

[B24] Kemman M. (2021). Trading Zones of Digital History. Berlin: De Gruyter.

[B25] Kim M. C. Zhu Y. Chen C. (2016). How are they different? A quantitative domain comparison of information visualization and data visualization (2000–2014). Scientometrics 107, 123–165. 10.1007/s11192-015-1830-0

[B26] Kim N. W. Im H. Riche N. H. Gajos K. Pfister H. (2018). Fostering Data Humanism With DataPortraits: Empowering People to Create a Personalized Visual Vocabulary. IEEE VIS Poster (VIS).

[B27] Kim N. W. Im H. Henry Riche N. Wang A. Gajos K. Pfister H. (2019). “DataSelfie: empowering people to design personalized visuals to represent their data” in Proceedings of the 2019 CHI Conference on Human Factors in Computing Systems (Glasgow Scotland Uk: ACM), 1–12.

[B28] Knebeler C. Scuto D. (2010). Belval: Passé, présent et avenir d'un site luxembourgeois exceptionnel (1911-2011). Esch-sur-Alzette: Le Phare.

[B29] Lankow J. Ritchie J. Crooks R. (2012). Infographics: The Power of Visual Storytelling. Hoboken, N.J: John Wiley and Sons, Inc.

[B30] Lanzing J. (1998). Concept mapping: tools for echoing the minds eye. J. Vis. Lit. 18, 1–14. 10.1080/23796529.1998.11674524

[B31] Lima M. (2014). The Book of Trees: Visualizing Branches of Knowledge. New York, NY: Princeton Architectural press.

[B32] Lockwood A. (1969). Diagrams: A Visual Survey of Graphs, Maps, Charts and Diagrams for the Graphic Designer. London; New York, NY: Studio Vista; Watson-Guptill.

[B33] Loukissas Y. A. (2019). All Data are Local: Thinking Critically in a Data-Driven Society. Cambridge, MA: The MIT Press. 10.7551/mitpress/11543.001.0001

[B34] Lupi G. (2017). Data Humanism: The Revolutionary Future of Data Visualization. PRINT Mag. Available online at: https://www.printmag.com/article/data-humanism-future-of-data-visualization/ (accessed January 10, 2023).

[B35] Manovich L. (2011). What is visualisation? Vis. Stud. 26, 36–49. 10.1080/1472586X.2011.548488

[B36] Mao Y. Wang D. Muller M. Varshney K. R. Baldini I. Dugan C. . (2019). How data scientists work together with domain experts in scientific collaborations: to find the right answer or to ask the right question? Proc. ACM Hum.-Comput. Interact. 3, 1–23. 10.1145/336111834322658

[B37] Miller G. W. (2020). The Exposome - A New Paradigm for the Environment and Health. Cambridge, MA: Academic Press.

[B38] Miller G. W. Jones D. P. (2014). The nature of nurture: refining the definition of the exposome. Toxicol. Sci. 137, 1–2. 10.1093/toxsci/kft251 24213143 PMC3871934

[B39] Novak J. D. Cañas A. J. (2008). The Theory Underlying Concept Maps and How to Construct and Use Them. Tech. Rep. IHMC CmapTools. Available online at: http://cmap.ihmc.us/Publications/ResearchPapers/TheoryUnderlyingConceptMaps.pdf (accessed September 05, 2023).

[B40] Nussbaumer Knaflic C. (2015). Storytelling With Data: A Data Visualization Guide for Business Professionals. Hoboken, NJ: Wiley.

[B41] Panagiotidou G. Gorucu S. Vande Moere A. (2020). Data badges: making an academic profile through a DIY wearable physicalization. IEEE Comput. Graph. Appl. 40, 51–60. 10.1109/MCG.2020.3025504 32956041

[B42] Panagiotidou G. Poblome J. Aerts J. Vande Moere A. (2022). Designing a data visualisation for interdisciplinary scientists. How to transparently convey data frictions? Comput. Support. Coop. Work CSCW. 31, 633–667. 10.1007/s10606-022-09432-9

[B43] Rhyne T. Tory M. Munzner T. Ward M. Johnson C. Laidlaw D. H. (2003). “Information and scientific visualization: separate but equal or happy together at last” in IEEE Transactions on Ultrasonics, Ferroelectrics and Frequency Control (Seattle, WA, USA: IEEE), 611–614.

[B44] Rogowitz B. E. Treinish L. A. (1998). Data visualization: the end of the rainbow. IEEE Spectr. 35, 52–59. 10.1109/6.736450

[B45] Romance N. R. Vitale M. R. (1999). Concept mapping as a tool for learning: broadening the framework for student-centered instruction. Coll. Teach. 47, 74–79. 10.1080/87567559909595789

[B46] Santoro C. Kirkland C. (2022). The warming stripes - Inspiring a movement. Nightingale Mag. 2022, 52–60.20067403

[B47] Schwabish J. A. (2021). Better Data Visualizations: A Guide for Scholars, Researchers, and Wonks. New York, NY: Columbia University Press.

[B48] Schwan H. Arndt J. Dörk M. (2022). Disclosure as a critical-feminist design practice for Web-based data stories. First Monday 27, 11. 10.5210/fm.v27i11.12712

[B49] Segel E. Heer J. (2010). Narrative visualization: telling stories with data. IEEE Trans. Vis. Comput. Graph. 16, 1139–1148. 10.1109/TVCG.2010.179 20975152

[B50] Silva S. Madeira J. Santos B. S. (2007). “There is more to color scales than meets the eye: a review on the use of color in visualization” in 2007 11th International Conference Information Visualization (IV'07) (Zurich, Switzerland: IEEE),943–950.

[B51] Silva S. Sousa Santos B. Madeira J. (2011). Using color in visualization: a survey. Comput. Graph. 35, 320–333. 10.1016/j.cag.2010.11.015

[B52] Steen G. (2018). Visual Metaphor: Structure and Process. Amsterdam; Philadelphia: John Benjamins Publishing Company.

[B53] Thrift N. J. (2008). Non-Representational Theory: Space, Politics, Affect. 1st ed. London: Routledge.

[B54] Tufte E. R. (1999). The Visual Display of Quantitative Information. 17th ed. Cheshire; Conn: Graphics Press.

[B55] Van Wijk J. J. (2006). Bridging the gaps. IEEE Comput. Graph. Appl. 26, 6–9. 10.1109/MCG.2006.120 17120907

[B56] Vora S. (2019). The Power of Data Storytelling. Thousand Oaks, CA: SAGE Publications India Pvt Ltd.

[B57] Ward M. O. (2008). “Multivariate Data Glyphs: Principles and Practice,” in Handbook of Data Visualization Springer Handbooks Comp.Statistics (Berlin, Heidelberg: Springer Berlin Heidelberg), 179–198.

[B58] Ware C. (2004). Information Visualization: Perception for Design. San Francisco, CA: Morgan Kaufman.

[B59] Wheeldon J. Faubert J. (2009). Framing experience: concept maps, mind maps, and data collection in qualitative research. Int. J. Qual. Methods 8, 68–83. 10.1177/160940690900800307

